# The Double-Sided Effect of Empowering Leadership on Constructive Voice Behavior: Focusing on the Mediating Effects of Task Significance and Task Overload

**DOI:** 10.3390/bs13020180

**Published:** 2023-02-16

**Authors:** Xueqin Tian, Heesun Chae

**Affiliations:** School of Business Administration, Pukyong National University, Busan 48513, Republic of Korea

**Keywords:** empowering leadership, constructive voice behavior, task significance, task overload, job characteristics

## Abstract

Focusing on job characteristics, this study examined the double-sided effect of empowering leadership on constructive voice behavior. We obtained and analyzed a total of 294 questionnaire responses from pairs of subordinates and supervisors in various industries in Korea. The results supported our hypotheses that task significance and task overload partially mediate the relationship between empowering leadership and constructive voice behavior. Specifically, we found that empowering leadership can promote constructive voice behavior by inducing a recognition of task significance and can suppress constructive voice behavior by causing task overload. These results confirm that empowering leadership indirectly influences constructive voice behavior through job characteristics. These findings have important theoretical and practical implications and highlight directions for future research.

## 1. Introduction

In today’s increasingly competitive business environment, companies must accurately identify and respond to changes in internal and external environments to maintain a competitive advantage [1]. In uncertain organizational contexts, the efforts of management alone are insufficient to ensure that companies survive and continue to develop. Employees’ discretional initiative to offer ideas for change and raise issues with existing work processes are essential. In particular, employee constructive voice behavior—the voluntary expression of constructive opinions, concerns, or ideas regarding work-related issues—greatly contributes to organizational innovation and successful adaptation to dynamic business environments [2,3,4,5,6].

While constructive voice behavior can be considered a unique type of Organizational Citizenship Behavior (OCB) [7]), it also carries potential risks and can give rise to challenges [6,8]. The risky nature of voice means that the individuals who engage in constructive voice behavior expose themselves to potential losses; when employees express their opinions and ideas, they can be seen as problem-causing individuals who are interfering with the *status quo* [6,9,10,11]. Thus, given the potential benefits and risks faced by employees who speak up, it is important to understand the factors that affect constructive voice behavior. In this regard, previous studies have identified the antecedent factors affecting constructive voice behavior, which include environmental factors (e.g., organizational culture, reward system) [12,13,14], individual factors (e.g., self-efficacy, personality temperament) [15,16,17], and social factors (e.g., fairness perception, trust, relationship with superiors, peer characteristics) [18,19,20]. In this study, we focused specifically on the influence of leaders−a social antecedent factor that predict constructive voice behavior.

Leader behavior is regarded as an important antecedent of employee constructive voice behavior because leaders influence workplace norms related to voice and thereby directly encourage or inhibit employee voice behavior [8]. In addition to being the most important targets of voice, leaders also significantly impact the outcomes of voice behavior, which can include job assignments, compensation, and performance evaluations [14]. In particular, empowering leadership, which is characterized by the delegation of authority and autonomy, can be considered closely related to constructive voice behaviors that aim to improve the work unit or organizational functioning [12,13,14]. However, just as every coin has two sides, empowering leadership has both positive and negative effects on organizational effectiveness [21,22,23,24]. In other words, responsibilities beyond the duties assigned by empowering leaders can cause employees to experience stress or tension, degrading job performance or causing job dissatisfaction [25,26,27]. Accordingly, studies have raised questions about whether empowering leadership leads exclusively to desirable outcomes. 

Cheong et al. [25] recently suggested that empowering leadership can have a double-sided effect on work role performance. In addition, Hao et al. [23] utilized a dualistic model of passion for showing that empowering leadership has impacts on both task performance and creative performance. However, these studies had limitations; specifically, they focused on individual cognitive and dispositional characteristics and did not consider the job characteristics that have been predicted to enhance willingness to exert effort. The core premise of the job characteristics model (JCM) [28] is that leaders can generate a willingness to exert effort by constructing the objective characteristics of a given job. Moreover, the fact that employees’ perceptions of the characteristics of their jobs vary means that the same empowering leadership can have different effects on employees’ attitudes and behaviors. In particular, we suggest that empowering leaders who share power with subordinates and emphasize autonomy can have an impact on the elements of the JCM, thereby affecting employees’ motivation (willingness to exert effort), which in turn affects their constructive voice behavior. Thus, recognizing the ambivalence of empowering leadership, we set out to analyze the dual effects of empowering leadership on constructive voice behavior by setting job characteristics as mediating variables, facilitating a more comprehensive evaluation of the effects of empowering leadership on individuals. 

In applying Job Characteristics Theory (JCR) [28] and Self Determination Theory (SDT) [29], we identified the job characteristic task significance as a mechanism through which empowering leadership can promote constructive voice behavior. Specifically, we examined the extent to which the responsibility and autonomy granted by empowering leaders to make employees aware of task significance and whether this awareness positively affects the constructive voice behaviors of employees who develop it. Meanwhile, drawing on Role Theory [30] and Resource Conservation Theory [31], we also analyzed the ways empowering leadership can inhibit constructive voice behavior. Beyond granting employees autonomy, empowering leaders to assign them additional responsibilities that can foster perceptions of task overload and cause employees to experience losses of resources because they have to invest more time and effort in performing these additional tasks and responsibilities. To make up for such losses, employees tend to reduce their proactive voice behaviors.

**Figure 1:**
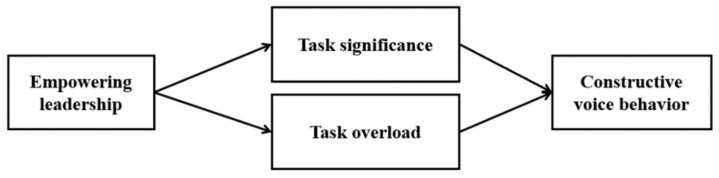
Research model.

In summary, we investigated the double-sided impacts of empowering leadership on constructive voice behavior by setting two job characteristics as mediating variables. Specifically, we examined whether empowering leadership promotes constructive voice behavior by boosting perceptions of task significance and whether it suppresses constructive voice behavior by boosting perceptions of task overload. Figure 1 shows the detailed research model.

## 2. Theoretical Background and Hypotheses

This section first reviews the theoretical background of constructive voice behavior and empowering leadership, then discusses the relationship between the positive side of empowering leadership and task significance, as well as the negative side of empowering leadership and task overload. After discussing previous studies, we propose our hypotheses.

### 2.1. Constructive Voice Behavior

Based on Exit-Voice-Loyalty Theory, Hirschman [32] defined voice as a response to dissatisfaction when problems arise and decline in firms, organizations, and states. Rusbult et al. [33] identified four categories of behavior—exit, voice, loyalty, and neglect—as general responses to dissatisfaction. Among them, voice can be viewed as an active and constructive response through which individuals attempt to revive and maintain satisfactory employment conditions. In other words, according to these scholars, voice encompasses the actions taken by employees who, rather than exiting their organizations, stay and draw attention to sources of dissatisfaction. 

Subsequently, Van Dyne et al. [34] described voice behavior as a type of extra-role behavior that entails challenging promotive characteristics to improve work organizations. Voice behavior emphasizes ideas and problems and has the characteristic of being change-oriented. At the same time, it implicitly carries the possibility of damaging relationships [35]. Specifically, Van Dyne et al. [36] regarded voice behavior as intentional motivation-based action that could be divided into pro-social voice, defensive voice, and acquiescent voice. Defensive voice involves expressing ideas that shift attention elsewhere based on fear; those who engage in defensive voice behavior propose ideas that focus on others to protect themselves. Meanwhile, an acquiescent voice entails expressing ideas that support the group rather than expressing one’s own opinions. Finally, pro-social voice is characterized by the cooperative expression of constructive ideas, information, or opinions to generate beneficial change in organizations. 

Liang et al. [10] further divided voice behavior into promotive voice behavior and prohibitive voice behavior, considering both its constructive and unsatisfactory content dimensions as well as its function as extra-role behavior. That study defined promotive voice as the expression of new ideas or suggestions that accompany innovative solutions and recommendations. In contrast, it defined prohibitive voice as the expression of concern about work practices, incidents, or employee behavior that focus on stopping or preventing harm. In other words, Liang et al. [10] viewed voice behavior as a positive and challenging concept that encompasses employees’ suggestions and expressions of concern regarding ways to improve the overall functioning of their work units or organizations. 

More recently, Maynes and Podsakoff [37] developed a new voice behavior framework that comprises behaviors that are both beneficial and harmful to organizations. They classified these behaviors into four dimensions: supportive, defensive, destructive, and constructive voice behaviors within the categories of promotive or prohibitive and challenge or preservation. This definition of constructive voice behavior emphasizes its challenging nature, indicating that it may harm interpersonal relationships and have negative consequences for employees who engage in it. It also contains the voluntary attributes of expressing ideas and opinions that have a facilitating and challenging nature and focus on functional changes to organizations. Table 1 summarizes these classifications of voice behavior.

In this study, we emphasize the characteristics through which constructive voice behavior leads to situations that involve benefits and risks for individuals. The proactive and pro-social nature of constructive voice behavior makes it an essential factor in driving organizational change and innovation [6,13,14]. However, because employees who engage in it aim to overcome the *status quo*—seeking to address past wrong decisions and identify problems in hopes of improving the current situation—they put themselves at risk [34,38]. Thus, to emphasize the two sides of empowering leadership, we focused on constructive voice behavior, which is a voluntary extra-role behavior that entails potential risks.

### 2.2. Constructive Voice Behavior

Empowering leadership is defined as a set of leader behaviors leaders use to share legitimate power with subordinates and give them levels of autonomy and responsibility that enable them to experience empowerment [21,39,40,41,42]. As a formal leadership behavior, empowering leadership expands the concept of psychological empowerment [22,41,43] to include enhancing the meaningfulness of work, encouraging subordinates to express opinions and ideas, fostering participation in decision-making, and facilitating information sharing and knowledge management [21,39,44].

A leader who engages in empowering leadership gives employees opportunities to derive meaning from their work by increasing their psychological empowerment and giving them self-determination in the performance of their tasks [22,26]. By instilling in employees the belief that they can perform their work and emphasizing their decision-making autonomy in work processes, empowering leaders positively affect the behaviors and attitudes of their employees [42,45].

Meanwhile, recent meta-analytic results have shown that empowering leadership has a negative or non-significant direct effect on certain outcome variables [26]. Indeed, scholars have found that empowering leadership is not advantageous in all organizational contexts, and not all employees are universally receptive to empowering initiatives from leaders [27]. While autonomy and the assignment of additional work were previously considered positive factors, they can also be burdens for employees, potentially contributing to role ambiguity [46]. The concept of the autonomy trap proposed by Langfred and Moe [47] applies in this context. In situations where individuals do not have adequate information to make decisions or where task technology requires high levels of organizational interdependence, the introduction of task autonomy may actually have overall negative effects [47]. Too much autonomy can impose levels of responsibility that employees are unwilling to bear. This greater degree of responsibility can induce insecurity in employees [48]. In sum, the effects of empowering leadership are complex and may have two distinct faces that warrant further investigation [22]. 

Seeking to explain the mixed perspectives and research findings regarding the impacts of empowering leadership, scholars have recently directed increasing attention to the duality or ambivalence of empowering leadership. Specifically, Cheong et al. [25] highlighted the duality of empowering leadership, interpreting its positive effects as enabling processes activated by self-efficacy and its negative effects as burdening processes. In a similar vein, Hao et al. [23] showed that empowering leadership has an indirect positive relationship with employee work performance and creative performance through harmonious passion and an indirect negative relationship with employee work performance through obsessive passion. These studies verified the approach’s ambivalent impact on job performance by examining individual characteristics but gave no consideration to job characteristics. Since empowering leadership is a variable related to the scope of responsibility and expansion of autonomy in task-related outputs, it also exerts a considerable influence on job perceptions.

Job characteristics are important in that they affect individuals’ psychological states, and psychological effects change individuals’ job attitudes and behaviors. In the context of job characteristics, leadership impacts employees’ perceptions of their jobs [49]. Meanwhile, Griffin [50] first verified the notion that leaders affect job perceptions without adjusting objective job characteristics; the subordinates in their experimental group received higher evaluations in key job characteristics even though their jobs underwent no substantial changes. Therefore, recognizing the ambivalence of empowered leadership, we set out to explain the relationship between empowering leadership and constructive voice behavior by setting two job characteristics as mediating variables.

### 2.3. Positive Side of Empowering Leadership

According to the JCR [28], individuals’ perceptions of the characteristics of their jobs impact their attitudes and behaviors. Fundamentally, the JCR posits that the five core elements—task significance, variety, autonomy, identity, and feedback—increase the likelihood of including positive psychological states in employees and result in positive work outcomes. Among the five core elements, task significance refers to the degree to which a job has an important effect on another person’s life or work. It leads to positive job performance by affecting key psychological states such as employees’ job meaning and sense of responsibility. When employees accurately understand the meaning of their tasks and feel that their work is important, their levels of interest in their jobs and intrinsic motivation increase. Prior research has shown that perceptions of task significance positively affect employee performance [51] and OCB [52].

Empowering leaders engage their subordinates in decision-making processes and give them opportunities to take control of their jobs by transferring authority [19,53]. In so doing, empowering leaders allows employees to develop a greater sense of task significance and exercise greater self-determination [54]. Task significance can provide the necessary ingredients for the effective development of employee challenge-oriented OCB by fostering positive affective states, satisfying psychological needs, and boosting intrinsic motivation [55,56]. High task significance enables employees to experience the positive impacts of their work on others, which is conducive to the realization of personal self-worth, stimulates employees’ sense of responsibility for their work, and makes employees view their work more positively. Such positive attitudes become sources of passion and energy for work [57], lead employees to actively devote themselves to their jobs, and increase their levels of outcomes [58,59]. Employees who feel a sense of task significance are likely to devote extra effort to the successful and timely completion of jobs, draw attention to the issues they find in work processes, express new ideas or solutions for how to improve the *status quo* and point out ways to do things better in the future [10]. In addition, perceived task significance alleviates psychological anxiety about mistakes or risks, thereby actively encouraging employees to suggest solutions to organizational problems and ways to improve current situations. In other words, the discretionary power granted through empowering leadership and participation in decision-making increases people’s awareness of task significance, which eventually promotes voluntary voice behavior. Based on the above discussion and previous studies, we posed the following hypothesis:

**Hypothesis** **1.** *Task significance mediates the relationship between empowering leadership and constructive voice behavior*.

### 2.4. Negative Side of Empowering Leadership 

Task overload refers to the perception among employees that they are overloaded with work relative to the time they have to perform their duties [30]. It occurs when employees are expected to take too much responsibility and complete too many tasks for the time, resources, and abilities they possess or when employees must devote too much energy to their roles [60]. As actual time spent in particular role activities increases, feelings of role overload increase. A heavy workload within a particular role can occupy one’s time and energy, thereby making it difficult to satisfy the demands of other work roles. Task overload forces employees to stretch their attention, effort, and resources thinly to fulfill overwhelming demands [61]. Prior research has shown a strong link between task overload and the degree of work intensity (i.e., very fast, tight deadlines, not enough time to complete work), demonstrating that it leads to work stressors, negative job attitudes, withdrawal, and absenteeism due to accidents [62]. 

According to Role Theory [63], employees experience role conflicts when the additional tasks and responsibilities they are assigned conflict with previously assigned roles. To resolve role conflicts and meet the needs of their conflicting or additional roles, employees must expend higher levels of effort than required to simply perform their existing roles [64,65,66]. By transferring authority to employees and engaging them in decision-making processes, empowering leaders gives employees additional roles and responsibilities. When the scope of employees’ roles and responsibilities expands in this way, they must perform more complex and difficult tasks. In other words, employees experience dual-task processing—a cognitive process that occurs when they must perform various tasks at the same time—between existing tasks and additional tasks [67], and this often causes them to experience task overload because they must devote more time and effort to meeting these needs.

Perceived task overload increases the physical and psychological stress experienced by employees [68]. The Conservation of Resources Theory [31] posits that since individuals tend to preserve, protect, and acquire resources, both perceived and actual losses of resources can cause psychological stress [69]. When they encounter threats to or actual loss of resources, individuals become defensive and attempt to protect their remaining resources [70,71]. Although the autonomy and authority gained through empowering leadership are also resources, the lost resources outweigh the acquired resources, causing individuals to eventually enter loss spirals in which the resources needed to offset the losses are rapidly reduced as the process repeats itself [72]. In the process of granting authority and autonomy, empowering leaders increases employees’ tasks and responsibilities. As a result, employees must invest more resources and effort in completing their tasks. This causes them to experience task overload, which they see as a loss of resources, and to protect their remaining resources for the future; they reduce unnecessary resource investment. In particular, to avoid unnecessary time and energy consumption, employees tend to minimize potentially challenging or dispute-related behaviors [6,73]. This causes them to reduce their constructive voice behavior because of its discretionary and voluntary extra-role nature.

The above discussion and previous studies suggest that employees working with empowering leaders tend to experience task overload, which suppresses their constructive voice behavior. Specifically, empowering leadership affects task overload, which negatively affects constructive voice behavior. Accordingly, we posited the following hypothesis:

**Hypothesis** **2.** *Task overload mediates the relationship between empowering leadership and constructive voice behavior.*

## 3. Methods

In this section, we explain the questionnaire survey we administered to test our hypotheses, describe our sample collection procedures, summarize the demographic characteristics of the sample, and present the questionnaire composition and measurement scale of the survey.

### 3.1. Sample and Procedures

To test our hypotheses, we conducted a questionnaire survey among employees of companies in various industries, including IT and construction, in the Republic of Korea. To avoid common method bias [74], we collected survey data from two different sources. We matched subordinates and their immediate supervisors one-to-one, distributing the subordinate questionnaire to subordinates and the supervisor questionnaire to supervisors. We used the supervisor questionnaire to measure the constructive voice behavior of subordinates, and the subordinate questionnaire included questions regarding supervisors’ empowering leadership, task significance, task overload, and control variables. All employees voluntarily participated in the survey, and it was announced in advance that the collected data would only be used for academic purposes. A total of 330 pairs of questionnaires were distributed, and 310 supervisor-subordinate dyads responded (response rate: 94%). After excluding those that did not answer sincerely or were not actually matched in supervisor-subordinate dyads, we used a total of 294 supervisor-subordinate dyads in the final analysis.

The final employee sample included 170 males (57.8%) and 124 females (42.2%). The age groups were 20s (19.7%), 30s (45.2%), 40s (25.2%), and 50s (9.9%). In terms of education level, 21 participants were high school graduates (7.1%), 54 were two-year college graduates (18.4%), 179 were four-year university graduates (60.9%), 38 had completed graduate school (12.9%), and 2 had completed schooling beyond graduate school (0.7%). In terms of organizational tenure, 139 participants (47.3%) had worked fewer than 5 years, 62 (21.1%) had worked more than 5 years and fewer than 10 years, 44 (15.0%) had worked fewer than 15 years, 19 (6.5%) had worked fewer than 20 years, and 30 (10.1%) had worked more than 20 years. The majority of the participants (143 employees, 48.6%) held office managerial positions. The remaining participants held positions in professional services (39 employees, 13.3%), research and development (38, 12.9%), production engineering (27, 9.2%), sales (19, 6.5%), and other duties (28 employees, 9.5%). Meanwhile, of the 294 supervisors, 58.8% were male, their average age was 42.16 (SD = 8.43), and their average organizational tenure was 12.51 (SD = 7.64). 

### 3.2. Measures

Excluding the control variables, participants responded regarding the study variables on a 7-point Likert scale (1 = not at all, 7 = very much). The focal employees measured independent and mediating variables, and their immediate supervisors measured dependent variables. The scale of the questionnaire adopts the reliability and validity recognized by previous studies. 

Empowering leadership was measured with 12 items (α = 0.96) adapted by Ahearne et al. [21]. Example questionnaire items include: “My leader helps me understand how my objectives and goals relate to that of the company” and “My leader helps me understand the importance of my work to the overall effectiveness of the company.” Constructive voice behavior was measured with six items (α = 0.71) used in Van Dyne and LePine [35]. Example questionnaire items include: “This subordinate develops and makes recommendations concerning issues that affect the work group” and “This subordinate speaks up and encourages others in our group to get involved in an issue that affect the group.” Task significance was measured with three items (α = 0.91) used in Hackman and Oldham [49]. Questionnaire items include: “My work is very important to the overall performance of the company”, “The results of my work are likely to significantly affect the lives or well-being of other people”, and “Many people are affected by my work performance.” Task overload was measured with three items (α = 0.89) developed by Bolino and Turnley [75]. Questionnaire items include: “The amount of work I am expected to do is too great”, “It often seems like I have too much work for one person to do”, and “I never seem to have enough time to get everything done at work.” We controlled for four employee demographic variables (gender, age, education, and organizational tenure) that research has shown affect constructive voice behavior [76]. This enabled us to clearly verify the relationship between the variables in this study. We measured gender as a dichotomous dummy variable coded as 0 for males and 1 for females. Meanwhile, we divided education into the following five categories: 1 = high school graduate, 2 = 2-year college graduate, 3 = 4-year university graduate, 4 = graduate school, and 5 = beyond graduate school. Finally, we measured age and organizational tenure in years.

## 4. Results

To measure the validity and reliability of variables, we performed exploratory factor analysis and Cronbach’s alpha coefficients. We derived descriptive statistics and correlation analysis results and verified the model fit using confirmatory factor analysis. In testing our hypotheses, we validated the mediation effect with AMOS 27.

### 4.1. Reliability and Validity Verification

We performed preliminary analyses to ensure there were no violations of linearity or homoscedasticity. The results indicated that the variance inflation factors (VIF) were all under 10. The Durbin-Watson value also fell within the acceptable range of 1.8–2.0 (close to the value of 2), implying that there was likely no autocorrelation problem in the data. 

Our tests to verify the reliability of each variable produced Cronbach’s alpha coefficients of 0.70 or higher for all variables, indicating high reliability [77]. Thus, we deemed the measurement items used in this study to have relatively high levels of internal consistency.

To verify the between-variable validity, we performed factor extraction using principal component analysis and exploratory factor analysis using the Varimax rotation method. Out of the four factors’ 24 items, we removed one with low factor loading (voice behavior 3) and adopted 23. All items showed factor loading values of 0.7 or more (0.733~0.890), so we judged them suitable for analysis [78]. The KMO value measuring the adequacy of the sample was 0.921, which is close to 1, and Bartlett’s sphericity test statistic, which verifies whether the correlation between variables is 0, was 6406.817 (df = 276, *p* = 0.000)—significant at the 0.001 level [78]. We, therefore, deemed the four variables to be clearly distinguished from one another and suitable for factor analysis. Table 2 shows the results of the reliability and factor analyses.

### 4.2. Descriptive Statistics and Correlation Analysis 

As shown in Table 3, we performed the descriptive statistical analysis and correlation analysis to investigate the correlations between the major variables. The correlation analysis showed that age, the control variable, had significant correlations with constructive voice behavior (r = 0.148, *p* < 0.05). It also showed that the independent variable, empowering leadership, had statistically significant correlations with the mediating variables, task significance (r = 0.455, *p* < 0.001) and task overload (r = 0.164, *p* < 0.01), and the dependent variable, constructive voice behavior (r = 0.367, *p* < 0.001). In addition, it revealed that task significance had a positive correlation (r = 0.280, *p* < 0.001) with constructive voice behavior. Finally, the analysis indicated that task overload had a non-significant negative correlation (r = −0.094, *ns*.) with constructive voice behavior. The relationships between all variables aligned with our hypotheses, and we subsequently reverified the correlations between them using regression analysis.

### 4.3. Confirmatory Factor Analysis (CFA) 

Prior to testing out hypotheses, we conducted CFA to assess the empirical distinctiveness of the study’s four variables. As Table 4 shows, the hypothesized measurement model produced an acceptable fit with the data (χ^2^[220] = 545.37, *p* < 0.001; CFI = 0.95, IFI = 0.95, RMR = 0.07). We found that the hypothesized four-factor structure fit significantly better than any alternative factor model. Thus, the results of the CFA supported the distinctiveness of the four study variables for subsequent analyses. 

### 4.4. Hypothesis Testing 

To verify the double-sided mediating effect of empowering leadership on constructive voice behavior, we used Amos 27.0. We set task significance and task overload as mediators operating in parallel and conducted a bootstrapping analysis to assess the indirect effects in the empowering leadership-constructive voice behavior relationship. 

As shown in Table 5, we found that empowering leadership had a significant effect on task significance [B = 0.480, 95% CI = (0.360, 0.586)] and task overload [B = 0.164, 95% CI = (0.043, 0.287)]. The analysis showed that task significance [B = 0.192, 95% CI = (0.057, 0.324)] and task overload [B = −0.203, 95% CI = (−0.304, −0.103)] had significant effects on constructive voice behavior, and the direct effect between empowering leadership and constructive voice behavior was significant [B = 0.330, 95% CI = (0.203, 0.465)]. 

As shown in Table 5, the effect size of them indicated that empowering leadership had an indirect linear effect on employees’ constructive voice behavior via task significance and task overload. After conducting 2000 bootstrap replicates, the effect size of task significance was calculated as 0.093 with a 95% bias-corrected confidence interval [0.024, 0.180], and the effect size of bootstrapping analysis of task overload was −0.034 with a 95% bias-corrected confidence interval [−0.076, −0.009]. The total effect size of the direct and indirect effects was 0.389 with a 95% bias-corrected confidence interval [0.282, 0.494]. These findings were significant because the bias-corrected confidence interval did not include zero. This analysis indicated that the association between empowering leadership and employees’ constructive voice behavior was mediated through employees’ task significance and task overload. Therefore, Hypotheses 1 and 2 were supported.

## 5. Discussion

In this section, we summarize our overall findings, discuss their theoretical and managerial implications, reveal the limitations of the current study, and outline directions for future research. 

### 5.1. Overall Findings

The purpose of the current study was to examine the dual effects of empowering leadership on constructive voice behavior through two job characteristics and to validate these effects with actual company data. The tests of our hypotheses confirmed that task significance and task overload partially mediated the relationship between the leaders’ empowering leadership and constructive voice behavior. 

### 5.2. Theoretical Implications

First, the current study contributes to research exploring the antecedent factors affecting the constructive voice behavior of employees. Researchers have examined the environmental, personal, and social factors that lead to voice behavior [6,8,11,12]. By elucidating the positive relationship between empowering leadership (a social factor) and constructive voice behavior, this study reaffirms the importance of empowering leadership in reinforcing employees’ constructive voice behavior. In so doing, it expands the flow of existing studies regarding the effects of various positive leadership styles, including transformational leadership [3], authentic leadership [79], and ethical leadership [80] on voice behavior.

Second, our findings shed new light on the contradictory relationship between empowering leadership and constructive voice behavior by revealing its paradoxical dual mechanisms, thereby contributing to the study of empowering leadership by showing a mix of positive and negative effects. Our in-depth approach enabled us to address the limitations of existing studies, which have mainly focused on the positive effects of empowering leadership and avoided its double-sided impacts. Cheong et al. [25] and Hao et al. [23] emphasized the dual mechanism-based effects of empowering leadership on job performance and creativity. In line with those studies, our findings highlight the heterogeneous and paradoxical mechanism-based effects of empowering leadership and provide empirical evidence of empowering leadership’s effectiveness.

Third, this is the first study to find that job characteristics have a mediating effect on the relationship between empowering leadership and employees’ constructive voice behavior. Previous studies have shown that empowering leadership can impact employees’ self-conceptions [81], social relationships [82], and psychological empowerment [83]. In addition, some studies have sought to identify the psychological mechanism between empowering leadership and voice behavior [84,85]. In contrast, we focused on job characteristics, which can create psychological ecosystems that foster internal motivation in employees, demonstrating that empowering leadership has divergent effects on employees’ constructive voice behavior because employees’ perceptions of the characteristics of their jobs vary. This integration of empowering leadership, Job Characteristics Theory, and constructive voice behavior have theoretical implications for future research. 

Fourth, we empirically examined the partial mediating effect of task significance on the relationship between empowering leadership and constructive voice behavior, finding that the actions of empowering leaders, such as transferring authority and granting higher levels of autonomy, can strengthen employees’ intrinsic motivation and enable them to derive greater meaning from their jobs. Subordinates recognize task significance when they feel that their jobs are meaningful and believe that they can significantly impact their organizations. Perceived task significance ultimately generates constructive voice behavior. To explain the positive effect of empowering leadership on constructive voice behavior, we used Job Characteristics Theory [28] and Self Determination Theory [29], which argue that autonomy enhances employees’ intrinsic motivation and thereby leads to positive outcomes. 

Fifth, we empirically examined the partial mediating effect of task overload on the relationship between empowering leadership and constructive voice behavior. According to Role Theory [63], empowering leaders assign additional roles and responsibilities to employees by transferring authority and involving them in decision-making processes, fostering perceived task overload in employees. Resource Conservation Theory [31] regards perceived task overload as a loss of resources, which prompts employees to conserve resources by avoiding further resource investment, such as constructive voice behavior. Our analysis showed that the increased autonomy resulting from empowering leadership does not always generate desirable results. Instead, we found that it can increase perceived task overload among employees and interfere with the positive relationship between empowering leadership and constructive voice behavior. These results suggest that empowering leadership also has negative effects—a finding that aligns with prior research showing that empowering leadership can burden and stress employees and thereby undermine job role performance [25]. 

### 5.3. Managerial Implications

Facilitating constructive voice behavior through leadership in organizations has become an increasingly important management practice. Our analysis confirmed the mediating effects of job characteristics on the relationship between empowering leadership and constructive voice behavior. This indicates that because employees have varying perceptions of the characteristics of their jobs, even if their leaders behave in the same ways, the effects of empowering leadership on constructive voice behavior may vary depending on job characteristics. 

Empowering leadership can have significant positive impacts on employees’ perceptions of their jobs. When leaders practice empowering leadership, employees’ perceived task significance tends to increase and influence their voice behavior. Leaders should therefore provide material resources and social support to increase employees’ job flexibility and autonomy and communicate with employees more about the meaning of their work. Doing so will increase employees’ perceived task significance and the sense of accomplishment they acquire from their jobs. In particular, in the case of core jobs that significantly impact organizational performance, continuously stimulating extra-role behaviors by motivating employees internally through empowering leadership is crucial. On the other hand, our analysis also showed that task overload negatively mediates the effect of empowering leadership on constructive voice behavior. This suggests that empowering leadership, through which leaders delegate authority and give employees more job autonomy, can cause employees to experience high levels of job-related pressure and stress. When leaders exercise empowering leadership, it may increase perceived task overload among employees, which can interfere with voluntary and discretionary voice behavior. Therefore, managers need to be aware that empowering leadership may not always produce desirable outcomes for their subordinates.

These ambivalent findings regarding empowering leadership may confuse managers seeking to encourage employees to express their ideas and opinions about important issues and provide channels and opportunities for voice delivery. Leaders with such aims should listen to employees and provide the support they need to identify and reduce the factors that can trigger the negative effects of empowering leadership. They should also endeavor to provide employees with continuous feedback regarding their performance on delegated responsibilities and authorities. By providing proper feedback, leaders can enable employees to respond more effectively to additional requirements and behave more proactively in the workplace [86]. Similarly, Hackman and Oldham [28] suggested that feedback positively impacts employees’ internal motivation and high-quality work performance. In addition, leaders must seek to understand the extent to which each employee will view additional responsibilities as a burden. For example, subordinates with a high need for growth tend to regard additional responsibilities as opportunities, while subordinates with a low need for growth tend to regard them as burdens. Therefore, companies should attempt to design leadership education programs that facilitate the positive effects of empowering leadership and establish sophisticated human resource management systems to support these programs. Meanwhile, organizations should also consider inviting managers to participate in executive education programs or leadership centers that provide training in effective empowering leadership behaviors.

### 5.4. Limitations and Future Research

The fact that we conducted a cross-sectional analysis at a specific point in time means that we could not avoid the influence of situational characteristics. To overcome this limitation, we administered questionnaires to both supervisors and subordinates. However, since we conducted a survey at a single point in time, it could not sufficiently reflect the sequential relationship between empowering leadership and constructive voice behavior. Future studies should seek to accurately gauge the causal relationship between these two variables by adopting longitudinal designs. 

While our analysis confirmed the mediating effects of task significance and task overload on the relationship between empowering leadership and constructive voice behavior, explaining this relationship more concretely would require a detailed examination of the relationship between individual characteristics and job characteristics or organization-related situational characteristics as moderating variables. For example, employees with proactive personalities or flexible role orientations will more actively accept and utilize the additional authority or autonomy granted by their superiors [87,88]. In short, we believe the scope of this study could be expanded by analyzing and testing the mutual influences of the relationships between job characteristics and individual characteristics or situational characteristics in three dimensions.

Developing an in-depth understanding of the relationship between empowering leadership and subordinates’ voice behavior will require exploration and verification of various mediating variables that we did not address in this study. For example, researchers could consider variables related to perceptions of emotions and harmonious and obsessive passion. When employees perceive themselves as released from bureaucratic constraints, they are more likely to develop positive affectivity for their work and view the expression of their opinions as meaningful, which will eventually lead them to engage in voice behavior.

Future studies should consider the effects of empowering leadership on a wider range of performance variables. For example, the impact of knowledge-sharing behavior, which is desirable behavior for organizations but a risk factor for individuals, would be an interesting topic for future research. Since knowledge-sharing behavior is a source of competitiveness, the moment employees share knowledge with their colleagues, they become a public good [89]. Such research would be especially important because empowering leaders encourages employees to share their know-how or skills with colleagues who need help.

Moreover, future research exploring the dark side of empowering leadership and examining the effects of each sub-dimension of empowering leadership on various types of voice behavior is crucial. For example, an examination of whether the four sub-dimensions (improving job meaning, promoting decision-making participation, expressing confidence in high performance, and providing autonomy from bureaucratic constraints) [21] of empowering leadership may have different effects on voice behavior could be very illuminating. Voice behavior can also be divided into four categories—supportive, defensive, destructive, and constructive voice behavior [37]—and future studies should seek to determine how each type relates to empowering leadership. Such research will increase the value and implications of the findings of this study.

## 6. Conclusions

Based on the JCR, the current study investigated the double-sided impacts of em-powering leadership on constructive voice behavior by setting two job characteristics as mediating variables in one framework. We found that empowering leadership positively impacts constructive voice behavior through task importance and negatively impacts constructive voice behavior through task overload. Despite this study’s limitations, we hope that its exciting findings will prompt other researchers to further deepen their scholarly understanding of empowering leadership and voice behavior in organizations.

## Figures and Tables

**Table 1:** Classifications of voice behavior.

Classification Criteria	Scholars	Contents
Exit–Voice–Loyalty Theory	Hirschman [32]	A response to dissatisfaction, an active and constructive behavior
A type of extra-role	Van Dyne et al. [34]	Challenging promotive characteristics
Motivation	Van Dyne et al. [36]	Pro-social voice, defensive voice, and acquiescent voice.
Content	Liang et al. [10]	Promotive voice behavior and prohibitive voice behavior
Function	Maynes & Podsakoff [37]	Supportive voice, defensive voice, destructive voice, and constructive voice

**Table 2:** Exploratory factor analysis and reliability analysis.

Factor	Measure	1	2	3	4
Empowering leadership	1. My leader helps me understand how my objectives and goals relate to those of the company	**0.851**	0.125	0.031	0.048
	2. My leader helps me understand the importance of my work to the overall effectiveness of the company	**0.857**	0.136	0.051	0.045
	3. My leader helps me understand how my job fits into the bigger picture	**0.872**	0.078	0.029	0.013
	4. My leader makes many decisions together with me	**0.849**	0.137	0.076	0.072
	5. My leader often consults me about strategic decisions	**0.797**	0.173	0.205	0.207
	6. My leader solicits my opinion about decisions that may affect me	**0.764**	0.198	0.266	0.108
	7. My leader believes that I can handle demanding tasks	**0.787**	0.207	0.316	0.061
	8. My leader believes in my ability to improve even when I make mistakes	**0** **.762**	0.172	0.266	0.035
	9. My leader expresses confidence in my ability to perform at a high level	**0** **.765**	0.126	0.280	0.131
	10. My leader allows me to do my job my way	**0** **.791**	0.071	0.186	−0.031
	11. My leader makes it more efficient for me to do my job by keeping the rules and regulations simple	**0** **.859**	0.117	0.034	−0.069
	12. My leader allows me to make important decisions quickly to satisfy customer needs	**0** **.731**	0.141	0.124	0.042
Voice	1. This subordinate develops and makes recommendations concerning issues that affect the work group	0.165	**0** **.892**	0.118	−0.041
	2. This subordinate speaks up and encourages others in this group to get involved in issues that affect the group	0.115	**0** **.847**	0.006	0.050
	4. This subordinate keeps well informed about issues where his/her opinion might be useful to this work group	0.227	**0** **.843**	0.070	−0.082
	5. This subordinate gets involved in issues that affect the quality of work life here in this group	0.148	**0** **.798**	0.152	−0.095
	6. This subordinate speaks up in this group with ideas for new projects or changes in procedures	0.198	**0** **.844**	0.111	−0.069
Task significance	1.My work is very important to the overall performance of the company	0.217	0.143	**0** **.862**	0.157
	2. The results of my work are likely to significantly affect the lives or well-being of other people	0.260	0.119	**0** **.877**	0.110
	3. Many people are affected by my work performance	0.251	0.127	**0** **.833**	0.132
Task overload	1. The amount of work I am expected to do is too great	0.075	−0.043	0.292	**0** **.836**
	2. It often seems like I have too much work for one person to do	0.110	−0.114	0.013	**0** **.889**
	3. I never seem to have enough time to get everything done at work	0.050	−0.049	0.095	**0** **.925**
Eigenvalue		9.989	3.330	2.574	1.516
% of variance		43.432	14.480	11.075	6.592
% of cumulative		43.432	57.912	68.987	75.579
Cronbach α value		0.960	0.918	0.911	0.885
KMO = 0.923, Bartlett (χ^2^ = 6361.150, df = 253, *p* =0.000)					

**Table 3:** Descriptive statistics and correlation analysis between variables.

Variable	Mean	S.D.	1	2	3	4	5	6	7
1. Age	37.122	8.415							
2. Gender	0.422	0.495	0.028						
3. Education	2.816	0.771	−0.052	−0.226 ***					
4. Tenure year	7.656	7.646	0.658 ***	−0.037	−0.077				
5. Task significance	4.804	1.049	0.120 *	−0.099	0.128 *	0.129 *			
6. Task overload	4.002	1.022	0.060	−0.029	0.154 **	0.119 *	0.288 ***		
7. Empowering leadership	4.800	1.066	0.081	−0.131 *	0.006	0.138 *	0.455 ***	0.164 **	
8. Constructive voice behavior	4.801	0.945	0.148^*^	−0.047	0.027	0.042	0.280 ***	−0.094	0.367 ***

N = 294, *: *p* < 0.05, **: *p* < 0.01, ***: *p* < 0.001.

**Table 4:** Confirmatory factor analysis.

Model	No. of Factors ^a^	χ^2^	df	Δχ^2^	RMSEA	CFI	IFI
Baseline model	4 factors: EL, TS, TO, CVB	545.37	220		0.07	0.95	0.95
Model 1	3 factors: (EL + TS), TO, CVB	1058.97	223	513.60 ***	0.11	0.87	0.87
Model 2	2 factors: (EL + TS + TO), CVB	1555.37	225	1009.00 ***	0.14	0.79	0.79
Model 3	1 factors: (EL + TS + TO + CVB)	2432.36	226	1886.99 ***	0.18	0.65	0.65

Note: EL = Empowering leadership; TS = Task significance; TO = Task overload; CVB = Constructive voice behavior; RMSEA = root mean square error of approximation; CFI = Comparative fit index, IFI = Incremental fit index.

**Table 5:** Mediation analysis results.

Effect	Standardized Estimate	BC 95% Confidence Interval	
		Lower	Upper
**Direct effect**			
EL → TS	0.480	0.360	0.586
TS → CVB	0.192	0.057	0.324
EL → TO	0.164	0.043	0.287
TO → CVB	−0.203	−0.304	−0.103
EL → CVB	0.330	0.203	0.465
**Indirect effect**			
EL → TS → CVB	0.093	0.024	0.180
EL → TO → CVB	−0.034	−0.076	−0.009
Total effect	0.389	0.282	0.494

Note: EL = Empowering leadership; TS = Task significance; TO = Task overload; CVB = Constructive voice behavior.

## Data Availability

Not applicable.
